# Application of High-Density DNA Resequencing Microarray for Detection and Characterization of Botulinum Neurotoxin-Producing Clostridia

**DOI:** 10.1371/journal.pone.0067510

**Published:** 2013-06-20

**Authors:** Jessica Vanhomwegen, Nicolas Berthet, Christelle Mazuet, Ghislaine Guigon, Tatiana Vallaeys, Rayna Stamboliyska, Philippe Dubois, Giulia C. Kennedy, Stewart T. Cole, Valérie Caro, Jean-Claude Manuguerra, Michel-Robert Popoff

**Affiliations:** 1 Institut Pasteur, Laboratory for Urgent Responses to Biological Threats, Paris, France; 2 Institut Pasteur, Epidemiology and Pathophysiology of Oncogenic Viruses, Paris, France; 3 CNRS, UMR3569, Paris, France; 4 Institut Pasteur, Anaerobic Bacteria and Toxins Unit, Paris, France; 5 Institut Pasteur, Genotyping of Pathogens and Public Health, Paris, France; 6 CNRS – CC093 Université Montpellier II, UMR5119 Ecosystèmes lagunaires, Montpellier, France; 7 Department of Biology II, University of Munich (LMU), Planegg-Martinsried, Germany; 8 Department of Research and Development, Veracyte, Inc., South San Francisco, California, United States of America; 9 Global Health Institute, Ecole Polytechnique Fédérale de Lausanne (EPFL), Lausanne, Switzerland; Cornell University, United States of America

## Abstract

**Background:**

*Clostridium botulinum* and related clostridia express extremely potent toxins known as botulinum neurotoxins (BoNTs) that cause severe, potentially lethal intoxications in humans. These BoNT-producing bacteria are categorized in seven major toxinotypes (A through G) and several subtypes. The high diversity in nucleotide sequence and genetic organization of the gene cluster encoding the BoNT components poses a great challenge for the screening and characterization of BoNT-producing strains.

**Methodology/Principal Findings:**

In the present study, we designed and evaluated the performances of a resequencing microarray (RMA), the PathogenId v2.0, combined with an automated data approach for the simultaneous detection and characterization of BoNT-producing clostridia. The unique design of the PathogenID v2.0 array allows the simultaneous detection and characterization of 48 sequences targeting the BoNT gene cluster components.

This approach allowed successful identification and typing of representative strains of the different toxinotypes and subtypes, as well as the neurotoxin-producing *C. botulinum* strain in a naturally contaminated food sample. Moreover, the method allowed fine characterization of the different neurotoxin gene cluster components of all studied strains, including genomic regions exhibiting up to 24.65% divergence with the sequences tiled on the arrays.

**Conclusions/Significance:**

The severity of the disease demands rapid and accurate means for performing risk assessments of BoNT-producing clostridia and for tracing potentials sources of contamination in outbreak situations. The RMA approach constitutes an essential higher echelon component in a diagnostics and surveillance pipeline. In addition, it is an important asset to characterise potential outbreak related strains, but also environment isolates, in order to obtain a better picture of the molecular epidemiology of BoNT-producing clostridia.

## Introduction

Botulism is a severe neuroparalytic disease caused by botulinum toxin, and characterized by acute descending flaccid paralysis. The disease is caused by consumption of food contaminated with pre-formed toxin (foodborne botulism), or by absorption of toxin produced *in situ* in wounds (wound botulism) or colonized intestinal tracts (infant/intestinal adult botulism) [1]. Botulinum neurotoxins (BoNT) are the most potent toxins known and are considered as one of the six highest risk threat agents of bioterrorism [2],[3].

BoNT are produced by six physiologically and genetically distinct bacteria, namely Clostridium botulinum Groups I to IV, and occasionally strains of C. butyricum and C. barati [4], [5]. These neurotoxin-producing bacteria can be further categorized in seven major toxinotypes (A, B, C, D, E, F and G) based on the antigenic properties of the toxins they produce [6],[7]. Toxinotypes A, B, E and more rarely F are responsible for human botulism cases, while infections by C and D toxinotypes are observed mainly in animals. Although most strains produce only one toxin, bivalent strains producing two different toxins (Ab, Af, Ba, and Bf) have also been reported [8]–[11]. In addition, *C. botulinum* strains producing a single toxin but carrying a silent gene for another (A(B)) [10], and strains producing a chimeric neurotoxin (C/D or D/C) have been described [12].

**Figure 1:**
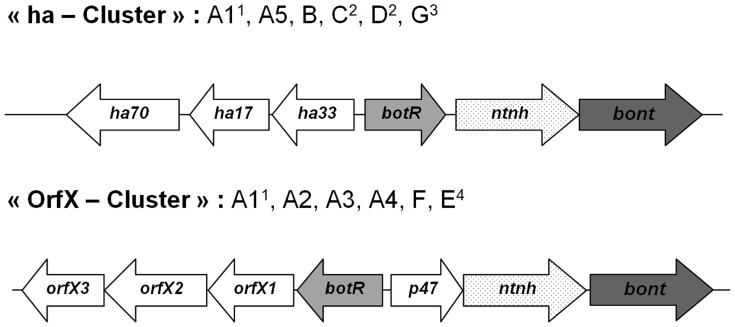
Neurotoxin gene cluster organization. In the gene cluster encoding the botulinum neurotoxin complex, the “ha cluster” appears to be associated with type A1, A5, B, C, D and G *bont* genes, and the “orf-X cluster” with type A1, A2, A3, A4, F and E *bont* genes. ^1^Although the type A1 *bont* gene is mostly found in a ha cluster, it has been found associated with an orf-X cluster in some single toxin strains and in all bivalent strains [18],[17].^2^The botR gene is in front of the ha genes in all toxinotype C and D strains.^3^The Ha33 gene is absent in all toxinotype G strains.^4^The botR gene is absent in all toxinotype E strains

The genes encoding the neurotoxins (*bont*) can be found on the chromosome, a plasmid or a bacteriophage, and are located in a cluster with other genes encoding associated non-toxic proteins (ANTPs) forming the botulinum neurotoxin complexes [13]. The recent sequencing of the *bont* genes has revealed significant sequence variations within the toxinotypes and has led to the recognition of several subtypes, with differences ranging between 2–32% at the amino acid level [14]. In addition, the BoNT gene cluster varies in structure and organization [5], leading to the differentiation of two different conserved cluster types, termed the “ha cluster” and the “orf-X cluster” (Figure 1). The “ha cluster” consists of a set of hemaglutinin (HA) genes while the “orf-X cluster” consists of a set of genes of unknown function called “*orf-Xs*” associated to the *bont* gene. The diversity in nucleotide sequence and genetic cluster organization can be explained by the occurrence of many recombination and insertion events [15], as well as *bont* gene transfer between some toxinotypes of *C. botulinum*, and with non-botulinum species [7].

This variability not only has implications for antibody neutralization strategies [14], but also poses a great challenge for screening and characterization of BoNT-producing strains. Sensitive laboratory detection techniques are necessary for establishing monitoring programs to track *C. botulinum* contamination in animals, food and environmental samples. In addition, characterization methods are essential for tracing potentials sources of contamination in outbreak situations or forensic investigations, and for performing molecular risk assessments of BoNT-producing clostridia. Moreover, characterization of genetic differences allows the development of improved diagnostics and therapeutic agents for the treatment of botulism [14]. In consequence, efforts have been centered on the development of molecular techniques allowing simultaneous detection and typing of *C. botulinum* toxinotypes, such as multiplex real time PCR and focused DNA microarray [16], [17] [17]–[19]. These assays are highly sensitive but rely on hybridization of sequence-specific primers for sufficient DNA amplification and/or on a limited number of probes for detection of target genomic regions, and may therefore be suboptimal when bacterial sequence divergence is high. In addition, although some multiplex assays allow simultaneous detection and differentiation of toxinotype A subtypes and strains [16], [20], they do not allow typing further than the toxin type or in-depth genetic characterization of strains belonging to other known toxinotypes. Several genotyping methods such as pulse-field gel electrophoresis, randomly amplified polymorphic DNA, multilocus variable number tandem repeat analysis and comparative genomic hybridization microarray have been applied to further differentiate strains within a toxinotype. However, such methods are often technically demanding, difficult to standardize between laboratories and sometimes produce poorly interpretable results, as subtype and strain discrimination relies on differences in observed electrophoretic or hybridization patterns [18], [19], [21]–[24]. In addition, these approaches often require high amounts of genomic DNA obtained from pure bacterial cultures. The use of high-yield random DNA amplification methods, combined to sequence-based technologies targeting multiple molecular markers, is indispensable to overcome these limitations and obtain a better overall resolution.

To overcome the problem of limiting amount of available DNA in biological samples, whole genome amplification (WGA) methods have been developed and successfully applied for a number of genotyping assays [25]–[29]. In addition, high-density resequencing microarrays (RMA) have emerged as a rapid detection and molecular characterization tool for a broad range of bacterial and viral agents [30],[31]. This technology has demonstrated reliable detection of sequences differing up to 10–15% from the prototype sequences on the array, enhancing the spectrum of detection [31],[32]. In order to determine a target nucleotide sequence, resequencing microarrays use closely overlapping (“tiled”) probe sets of 25 mers, which contain one perfectly matched and three mismatched probes per base for both strands of the target genes [33]. In the present study, we evaluated the PathogenID v2.0 resequencing microarray, containing probes for the detection and characterization of neurotoxin-producing *Clostridium* species.

## Results

### Design of PathogenID microarray for *Clostridium* detection and characterization

Detailed description of prototype sequences selected for *Clostridium* detection and characterization is shown in table 1. The array contains 4 tiled sequences targeting housekeeping genes of *Clostridium* species: the complete *rrs* gene of *C. botulinum* toxinotype A and partial *rpoB* genes of *C. difficile*, *C. perfringens* and *C. botulinum* toxinotype A. In addition, the RMA includes 48 tiled sequences ranging from 149 to 200 nt in length, targeting representative botulinum neurotoxin gene cluster components (*bont*, *ntnh*, *botR*, *ha17*, *ha70*, *ha33*, *orf-X1*, *orf-X2*, *orf-X3* and *p47*) of toxinotypes A through G, as well as three toxinotype A subtypes (A1, A2 and A3). Finally, it contains 19 tiled regions of 200 nt in length for the detection and characterization of other specific toxins produced by *C. tetani*, *C. difficile*, *C. perfringens*, *C. sordellii*, *C. septicum* and *C. oedematiens*.

**Table 1:** Description of the sequences tiled on the PathogenID v2.0 microarray for the detection and characterization of BoNT-producing clostridia.

Tiled sequence description		Species	Type	Strain	Genbank accession no.	Position (nt)	length (nt)
**Housekeeping genes**							
rrs	16S rRNA	C. botulinum	A1	DSM1734	X73442.1	7..1513	1506
rpoB	RNA polymerase (subunit β)	C. botulinum	A1	NCTC7272	Y16466.1	1249..1755	506
rpoB	RNA polymerase (subunit β)	C. perfringens		ATCC13124	CP000246.1	2986532..2986026	506
rpoB	RNA polymerase (subunit β)	C. difficile		630	AM180355.1	91744..92253	509
**Neurotoxin complex genes**							
bont/A1	Botulinum neurotoxin type A1	C. botulinum	A1	Hall	AF461540.1	11250..11450	200
bont/A2	Botulinum neurotoxin type A2	C. botulinum	A2	Kyoto	X73423.1	1771..1971	200
bont/B1	Botulinum neurotoxin type B1	C. botulinum	B1	Okra	AB232927.1	14210..14410	200
bont/Bnp	Botulinum neurotoxin type Bnp	C. botulinum	Bnp	ATCC25765	X71343.1	3586..3786	200
bont/C1	Botulinum neurotoxin type C1	C. botulinum	C1	468	X53751.1	1876..2076	200
bont/D	Botulinum neurotoxin type D	C. botulinum	D	1873	X54254.1	1697..1897	200
bont/E	Botulinum neurotoxin type E	C. botulinum	E1	Beluga	X62089.1	1213..1413	200
bont/F	Botulinum neurotoxin type F	C. botulinum	F6	202	M92906.1	606..806	200
bont/G	Botulinum neurotoxin type G	C. botulinum	G	NCFB3012	X74162.1	1651..1851	200
ntnh/A1	Non-toxigenic non-hemagglutinin	C. botulinum	A1	Hall	AF461540	8501..8695	194
ntnh/A2	Non-toxigenic non-hemagglutinin	C. botulinum	A2	Kyoto	X87974	2453..2647	194
ntnh/A3	Non-toxigenic non-hemagglutinin	C. botulinum	A3	Mascarpone	DQ310546	5690..5884	194
ntnh/Bp	Non-toxigenic non-hemagglutinin	C. botulinum	B1	Okra	AB232927	9649..9843	194
ntnh/C	Non-toxigenic non-hemagglutinin	C. botulinum	C1	468	X72793	4757..4966	209
ntnh/D	Non-toxigenic non-hemagglutinin	C. botulinum	D	1873	AB012112	4483..4692	209
ntnh/E	Non-toxigenic non-hemagglutinin	C. botulinum	E	Mashike	D12697	2644..2838	194
ntnh/Ebut	Non-toxigenic non-hemagglutinin	C. butyricum	E4	BL6340	D12739	2644..2838	194
ntnh/F	Non-toxigenic non-hemagglutinin	C. botulinum	F6	202	S73676	2664..2858	194
ntnh/G	Non-toxigenic non-hemagglutinin	C. botulinum	G	ATCC27322	X87972	3390..3599	209
ha17/A	Hemagglutinin 17	C. botulinum	A1	Hall	AF461540	4022..3822	200
ha17/Bp	Hemagglutinin 17	C. botulinum	B1	Okra	AB232927	5170..4970	200
ha17/C	Hemagglutinin 17	C. botulinum	C1	468	X72793	3181..2981	200
ha17/D	Hemagglutinin 17	C. botulinum	D	1873	AB012112	2907..2707	200
ha17/G	Hemagglutinin 17	C. botulinum	G	ATCC27322	X87972	1749..1600	149
ha70/A	Hemagglutinin 70	C. botulinum	A	Hall	AF461540	3166..3005	161
ha70/Bp	Hemagglutinin 70	C. botulinum	B1	Okra	AB232927	4314..4153	161
ha70/C/D	Hemagglutinin 70	C. botulinum	C1	468	X72793	2337..2173	164
ha70/G	Hemagglutinin 70	C. botulinum	G	ATCC27322	X87972	945..787	158
ha33/A	Hemagglutinin 33	C. botulinum	A1	Hall	AF461540	4603..4403	200
ha33/Bp	Hemagglutinin 33	C. botulinum	B1	Okra	AB232927	5748..5548	200
ha33/C/D	Hemagglutinin 33	C. botulinum	C1	468	X72793	3750..3550	200
orfX1/A2	Orf-X component 1	C. botulinum	A2	Kyoto	AB004778	524..315	209
orfX1/A3	Orf-X component 1	C. botulinum	A3	Mascarpone	DQ310546	1001..792	209
orfX1/E	Orf-X component 1	C. botulinum	E	Iwanai	D88418	1046..837	209
orfX2/A2	Orf-X component 2	C. botulinum	A2	Kyoto	AY497358	4619..4373	246
orfX2/E	Orf-X component 2	C. botulinum	E	Iwanai	D88418	256..10	246
orfX3/A2	Orf-X component 3	C. botulinum	A2	Kyoto	AY497358	2445..2236	209
botR/A1	Transcriptionnal regulator BotR	C. botulinum	A1	Hall	AF461540	5502..5702	200
botR/A2	Transcriptionnal regulator BotR	C. botulinum	A2	Kyoto	X96493	229..29	200
botR/A3	Transcriptionnal regulator BotR	C. botulinum	A3	Mascarpone	DQ310546	1438..1238	200
botR/B	Transcriptionnal regulator BotR	C. botulinum	B1	Okra	AB232927	6653..6853	200
botR/C	Transcriptionnal regulator BotR	C. botulinum	C1	468	X72793	512..712	200
botR/D	Transcriptionnal regulator BotR	C. botulinum	D	1873	AB012112	238..438	200
botR/G	Transcriptionnal regulator BotR	C. botulinum	G	ATCC27322	X87972	2595..2795	200
p47/A2	P 47	C. botulinum	A2	Kyoto	X96493	779..937	158
p47/A3	P 47	C. botulinum	A3	Mascarpone	DQ310546	1976..2134	158
p47/E	P 47	C. botulinum	E	Iwanai	D88418	1881..2081	200
p47/F	P 47	C. botulinum	F6	202	Y10770	946..1116	170
**Clostridial toxin genes**							
C2	C2 toxin (component 1)	C. botulinum		(C)-203U28	D88982.1	838..1038	200
C2	C2 toxin (component 2)	C. botulinum		(C)-203U28	D88982.1	2245..2445	200
tent	Tetanus neurotoxin	C. tetani		Massachusetts	X04436.1	2026..2226	200
toxB	Toxin B	C. difficile		VPI10463	X53138.1	1518..1718	200
toxB	Toxin BF	C. difficile		1470	Z23277.1	1183..1383	200
toxA	Toxin A	C. difficile		VPI10463	X51797.1	2521..2721	200
cpa	α-toxin	C. perfringens		S13	L43546.1	1317..1517	200
cpb1	β-toxin	C. perfringens		NCTC 8533	L13198.1	510..710	200
cpb2	β2-toxin	C. perfringens	Porcine	CWC245	L77965.1	418..618	200
cpb2	β2-toxin	C. perfringens	Equine	D21/98	AJ537535.1	199..399	200
cpb2	β2-toxin	C. perfringens	Bovine	JGS4147	AY609175	151..351	200
etxD	ε-toxin D	C. perfringens		NCTC 8346	M95206.1	714..914	200
pfoR	τ-toxin (perfringolysin)	C. perfringens		lambda gt10	M81080.1	3529..3729	200
enterotoxin	Enterotoxin	C. perfringens		NCTC8239	M98037.1	787..987	200
toxin-iota-a	ι-toxin (component Ia)	C. perfringens		NCIB10748	X73562.1	2254..2454	200
toxin-iota-b	ι-toxin (component Ib)	C. perfringens		NCIB10748	X73562.1	3515..3715	200
toxin-LT82	Lethal toxin 82	C. sordellii		82	X82638.1	1595..1795	200
α-toxin	α-toxin	C. septicum		NCTC547	D17668.1	1062..1262	200
α-toxin	α-novyi toxin	C. oedematiens		ATCC19402	Z48636.1	1286..1486	200
**Quinolone-resistance determining regions**							
gyrA-qrdr	Gyrase (subunit A)	C. perfringens		ATCC13124	CP000246.1	7269..7466	197
gyrA-qrdr	Gyrase (subunit A)	C. difficile		630	AM180355.1	6231..6428	197
parC-qrdr	Topoisomerase (subunit IV)	C. perfringens		SM101	CP000312.1	2254972..2255160	188

### Resequencing of *Clostridium* with the PathogenID v2.0 microarray

**Figure 2:**
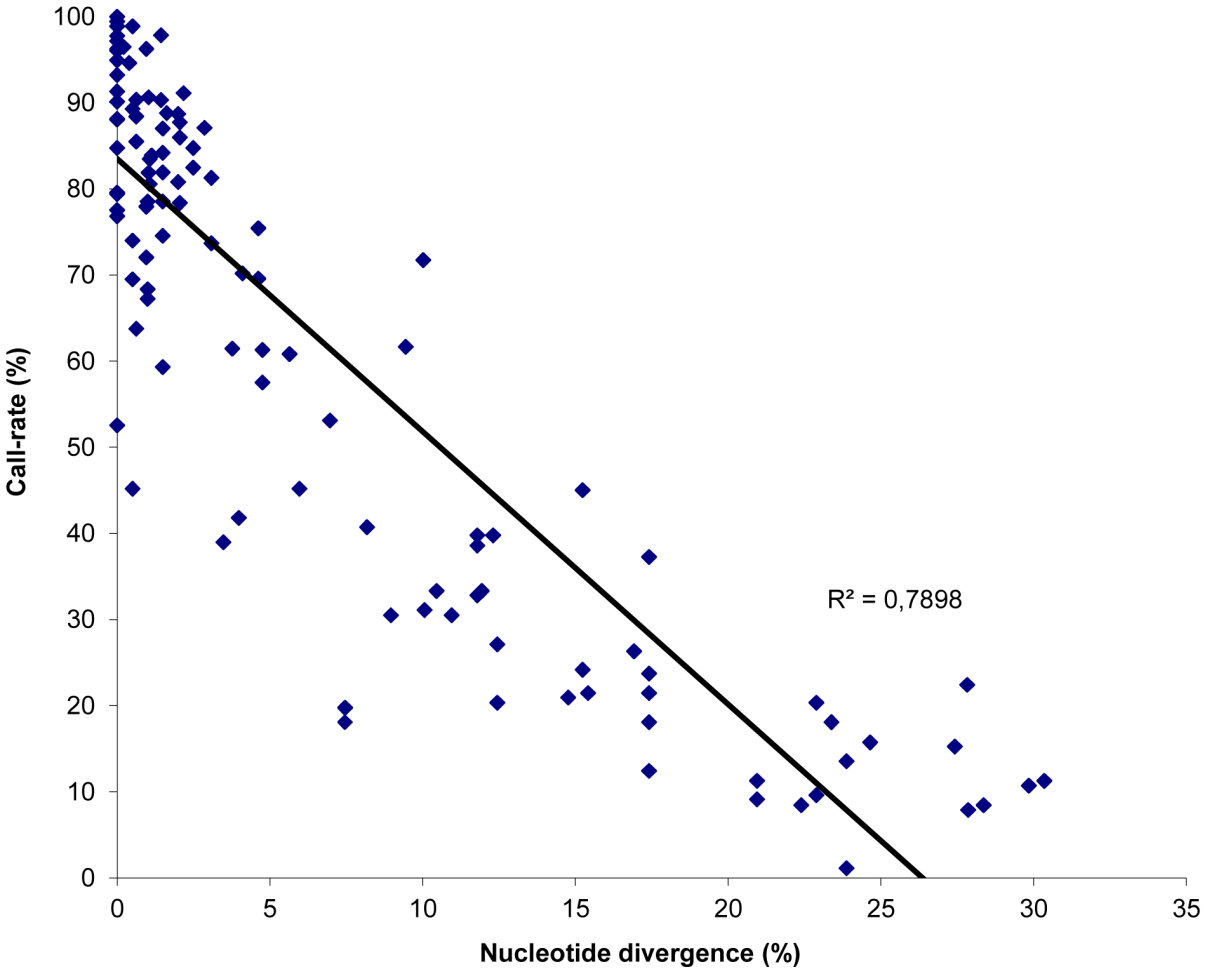
Detection spectrum of the PathogenID_v2.0 according to the nucleotide diversity of the tested BoNT-producing strains. The spectrum of detection of BoNT-producing clostridia by the PathogenID v2.0 was assessed using 9 well-characterized strains representative of all toxinotypes and several toxin A subtypes. Each sequence obtained by the RMA for which the percentage of nucleotide divergence compared to the corresponding tiled sequence is known (*n* = 118), is indicated by a blue diamond, and presented according to the percentage of nucleotide bases determined by the RMA (call rate). The linear association between these two parameters is shown, and demonstrates a good correlation (correlation coefficient R^2^ of 0.79).

The ability of the PathogenID v2.0 prototype sequences to detect and subtype neurotoxin-producing *C. botulinum* strains was assessed by performing a blind analysis of genomic DNA from eleven well-characterized strains representative of the main toxinotypes and subtypes. The observed call-rate for each tiled sequence is detailed in table 2. The call-rates marked in bold indicate sequences retained after filtering using the defined threshold. The average base call rate was 91.6% (range 52.5–100%) for strains for which genome sequence was identical to the tiled sequences. The analysis of more distant genomic sequences showed that these were successfully retrieved by RMA (Figure 2), and retained after filtering when they presented up to 24.7% divergence. The mean resequencing accuracy (correctly re-sequenced bases in comparison to reference sequencing results or database sequences) was 98.9% (range 87.1–100.0%). For all strains analysed by the RMA and the automated filtering approach, at least one sequence targeting a housekeeping gene and one sequence targeting a neurotoxin gene cluster component were successfully retrieved. For all bivalent strains analysed by RMA, two distinct *bont* gene prototype sequences could be retrieved: bont/B and/F sequences for strain 168.08 (toxinotype Bf2), and bont/A and/B sequences for strains NCTC 2916 [toxinotype A1(B)] and 1430-11 [toxinotype A5(B′)]. Additionally, sequences of two complete sets of ha and orf-X cluster components were retrieved for strains 168.08 and NCTC 2916, but as expected only sequences of ha cluster components were recovered for strain 1430-11. Finally, the RMA did not only allow the retrieval of sequences specifically targeting BoNT gene cluster components *antp* genes from the toxinotype of the strain analysed, but also sequences targeting *antp* genes from other toxinotypes belonging to the same taxonomic group. In particular, toxinotype B prototype sequences (e.g. botR/B, ha17/B) were successfully retrieved for strain Hall (toxinotype A), although these sequences show up to 11% divergence with the corresponding regions of the Hall strain genome. Similarly, prototype sequences targeting toxinotype A1 *antp* genes were retrieved for the BL6 strain (toxinotype B), and prototype sequences targeting toxinotypes A2 and A3 *antp* genes were retrieved for the NCIB10658 strain (toxinotype F). This was also observed for strains belonging to the *C. botulinum* taxonomic group III strains, as both toxinotype C and toxinotype D *antp* sequences were retrieved for strains 468 and 1873.

**Table 2:** Call-rates observed for the different *C. botulinum* sequences retrieved by the PathogenID V2.0 microarray for all tested bacterial strains.

Tiled C. botulinum sequence	Clostridium botulinum toxin subtype and strain										
	A1	A5(B′)	A1(B)	A3	B2	Bf2	F6	E1	C1	D	G
	Hall	1430.11	2916	Loch Maree	BL6	168.08	NCIB 10658	K16	468	1873	NCIB 10714
**Housekeeping genes**											
rrs A1	**88,1**	**83,5**	**83,9**	**80,6**	**90,7**	**21,5**	**85,1**	**69,8**	**71,7**	**61,7**	**69,2**
RpoB A1	**96,5**	**96,3**	**94,6**	**91,1**	**90,7**	**90,7**	**95,2**	16,4	**23,6**	**15,7**	**16,4**
**Neurotoxin genes**											
bont/A1	**97,2**	**87,0**	**97,7**	23,7	6,2	8,5	11,9	7,3	16,4	11,3	7,9
bont/A2	18,1	12,4	21,5	**88,7**	8,5	9,0	16,4	7,9	7,9	2,3	7,9
bont/B1	14,7	**18,1**	**78,5**	11,3	**19,8**	**88,7**	11,3	10,2	13,6	1,7	10,7
bont/Bnp	10,2	**27,1**	**35,6**	10,7	**20,3**	**35,6**	10,2	3,4	8,5	2,8	11,3
bont/C1	14,1	8,5	10,7	14,1	4,5	7,3	16,9	10,7	**99,4**	1,1	11,9
bont/D	13,6	2,8	12,4	21,5	4,5	5,7	19,2	15,3	13,6	**84,7**	12,4
bont/E	6,8	1,7	2,8	9,6	4,5	1,1	6,2	**67,2**	5,6	0,6	2,3
bont/F	16,4	4,0	16,9	23,7	11,9	**49,2**	**53,1**	11,3	13,6	3,4	8,5
bont/G	11,9	7,9	11,9	11,3	5,6	9,0	11,3	8,5	10,2	3,4	**76,8**
**Non-toxin non-hemagglutinin gene**											
ntnh/A1	**99,4**	**81,9**	**90,6**	**75,4**	10,5	**78,4**	**55,6**	9,4	8,2	1,2	6,4
ntnh/A2	**86,0**	**78,4**	**87,7**	**60,8**	15,8	**76,6**	**80,7**	13,5	7,6	1,2	8,2
ntnh/A3	**70,2**	**73,7**	**81,3**	**69,6**	8,8	**81,3**	**48,5**	8,2	7,0	0,6	7,0
ntnh/Bp	11,1	2,3	14,6	12,3	**97,1**	**98,2**	19,9	8,8	20,5	4,1	**29,2**
ntnh/C	26,9	11,8	17,2	17,7	16,1	9,7	13,4	14,5	**96,2**	**72,0**	9,7
ntnh/D	**32,8**	**21,0**	24,2	19,4	22,0	**17,2**	13,4	14,5	**96,2**	**79,6**	9,1
ntnh/E	21,1	12,3	24,0	**26,3**	7,6	9,9	15,8	**87,1**	15,8	2,3	11,1
ntnh/Ebut	21,6	11,7	24,0	26,3	6,4	9,9	15,2	**86,5**	15,2	1,8	11,1
ntnh/F	**45,0**	**38,6**	**39,8**	**39,8**	9,4	**43,3**	**33,3**	22,8	14,0	1,8	9,4
ntnh/G	27,4	14,5	22,6	22,0	16,1	15,1	14,0	7,0	24,2	5,9	**64,5**
**ha-cluster components**											
ha70/A	**94,9**	**63,8**	**91,3**	13,0	**76,8**	**96,4**	9,4	3,6	9,4	0,0	7,2
ha70/Bp	**88,4**	**77,5**	**85,5**	13,8	**84,8**	**92,0**	6,5	3,6	8,0	0,7	8,0
ha70/C/D	7,8	4,3	7,1	13,5	5,7	7,1	5,7	5,7	**100,0**	**79,4**	6,4
ha70/G	14,8	23,0	15,6	10,4	23,0	11,9	12,6	5,2	25,2	5,2	**45,9**
ha17/A	**98,9**	**80,8**	**97,2**	15,8	**86,4**	**96,6**	15,8	11,3	19,8	8,5	11,9
ha17/Bp	**84,7**	**84,2**	**82,5**	15,8	**84,2**	**84,2**	14,7	13,0	19,2	9,6	13,0
ha17/C	10,7	7,9	5,6	18,1	8,5	7,3	10,2	7,3	**100,0**	**89,3**	7,9
ha17/D	11,3	8,5	4,5	16,4	8,5	6,8	7,9	6,2	**100,0**	**84,7**	9,0
ha17/G	11,1	3,2	4,8	9,5	3,2	5,6	5,6	6,3	9,5	0,0	**57,9**
ha33/A	**96,0**	**45,2**	**78,5**	14,1	**49,2**	**85,3**	8,5	6,2	13,6	4,0	9,0
ha33/Bp	**30,5**	**30,5**	**33,3**	7,3	**55,4**	**41,8**	6,8	8,5	6,8	2,3	4,5
ha33/C/D	19,2	5,1	15,3	10,7	9,0	4,0	10,7	7,9	**99,4**	0,6	9,0
botR/A1	**93,2**	**59,3**	**74,6**	18,1	**83,6**	**89,3**	13,6	5,1	16,4	0,6	10,7
botR/Bp	**81,9**	**68,4**	**88,1**	20,3	**67,2**	**93,2**	13,0	7,3	17,5	0,6	10,7
botR/C	7,9	5,1	14,1	11,3	9,6	13,6	14,1	7,3	**98,9**	**45,2**	7,3
botR/D	9,0	6,8	13,6	11,3	8,5	**11,9**	15,3	7,3	**98,9**	**52,5**	8,5
botR/G	15,8	4,5	11,9	11,3	9,0	2,3	7,9	4,5	13,0	0,6	**46,9**
***orfX*** **-cluster components**											
orfX1-A2	5,4	1,1	**57,5**	**90,3**	6,5	**59,7**	**73,7**	3,8	7,5	3,8	6,5
orfX2-A2	14,8	4,5	**90,1**	**88,8**	9,0	**68,2**	**83,9**	11,2	10,8	6,7	12,6
orfX3-A2	9,7	2,2	**87,1**	**97,8**	10,8	**96,8**	**86,6**	14,0	20,4	7,5	9,7
orfX1-A3	8,6	5,4	**78,0**	**61,3**	7,5	**41,9**	**54,3**	5,4	7,5	1,6	5,9
orfX1-E	6,5	5,9	9,1	11,3	3,2	9,1	7,5	**68,3**	8,1	7,5	6,5
orfX2-E	9,9	5,4	15,2	22,4	6,3	7,2	17,0	**71,3**	10,3	1,8	7,2
botR/A2	9,6	1,1	**39,0**	**74,0**	8,5	**62,1**	**42,9**	2,8	10,2	2,3	4,5
botR/A3	8,5	0,6	**68,4**	**41,8**	2,8	**69,5**	**39,5**	5,1	10,2	1,7	6,2
p47/A2	9,6	3,7	**40,7**	**31,1**	6,7	36,3	36,3	6,7	14,1	1,5	8,1
p47/A3	10,4	1,5	**90,4**	**61,5**	6,7	**69,6**	**72,6**	5,9	15,6	1,5	7,4
p47/E	5,1	1,1	**21,5**	**33,3**	2,3	23,2	**19,8**	**80,8**	4,0	0,0	2,3
p47/F	8,8	0,7	8,8	6,1	4,8	**95,9**	19,0	6,1	8,8	1,4	12,9

Sequences retained after filtering using the defined threshold are designated in bold.

### Automated filtering and taxonomic identification of resequencing results

Automated filtering by TaxFinder using the defined parameters retained between 7 and 27 *C. botulinum* sequences for each strain (Table 2). Of the retained sequences, 98.8% (168/170) allowed retrieval of at least one BLAST hit, and 99,4 % (167/168) of the retrieved BLAST hits lead to correct taxonomic identification at least to the toxinotype level. Moreover, blasting of all remaining unfiltered *C. botulinum* prototype sequences (n = 358) did not return any BLAST hit, indicating that no useful sequence was excluded. For all reference strains the systematic BLAST strategy of retained sequences obtained by the RMA allowed successful identification of the clostridial species and taxonomic group using housekeeping genes prototype sequences (Table 3). The strategy also permitted in-depth characterization of all tested strains using the BoNT gene cluster prototype sequences (Table 3). Moreover, BLAST analysis allowed simultaneous typing of the neurotoxin up to the subtype level, as for each strain a unique best hit or multiple best bacterial hits were retrieved and corresponded to the correct subtype. Finally, the turnaround time of this automated approach was inferior to 2 hours, extensively decreasing the time needed for filtering of sequences, blasting of retained sequences and result analysis.

**Table 3:** Identification and characterization of *C. botulinum* strains based on the sequences retrieved by the PathogenID v2.0 microarray and the BLAST analysis results.

Strain	Toxin subtype	PathogenID v2.0 RMA results		
		Taxonomic identification	Neurotoxin subtype	*BoNT* gene cluster characterization
Hall	A1	*C. botulinum* group I	A1	ha+/OrfX−
1430.11	A5(B′)	*C. botulinum* group I	A5, B	ha+/OrfX−
2916	A1(B)	*C. botulinum* group I	A1, bivalent B	ha+/OrfX+
Loch Maree	A3	*C. botulinum* group I	A3	ha-/OrfX+
BL6	B2	*C. botulinum* group I	proteolytic B	ha+/OrfX−
168.08	Bf2	*C. botulinum* group I	bivalent B, F2	ha+/OrfX+
NCIB10658	F6	*C. botulinum* group I	proteolytic F	ha-/OrfX+
K16	E1	*C. botulinum* group II	E1	ha-/OrfX+
468	C1	*C. botulinum* group III	C1	ha+/OrfX−
1873	D	*C. botulinum* group III	D	ha+/OrfX−
NCIB10714	G	*C. botulinum* group IV	G	ha+/OrfX−

### Identification, typing and characterization of a foodborne botulism outbreak strain

**Figure 3:**
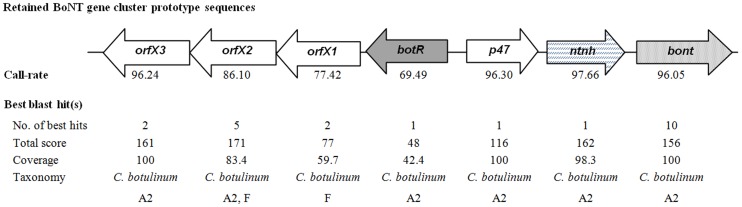
*C. botulinum* sequences retrieved by the PathogenID_v2.0 in contaminated food and BLAST analysis results. A schematic representation of the BoNT gene cluster sequences obtained by the RMA for the enrichment culture of the contaminated salad specimen is shown. The orientation and arrangement of these components were reproduced after Franciosa *et* al [59]. For each component, the highest call-rate observed and the results of the best retrieved BLAST hit(s) (defined as the BLAST hit demonstrating the highest total score) are given i.e. the number of best hits retrieved, as well as the corresponding query sequence coverage, total score, and taxonomy.

**Figure 4:**
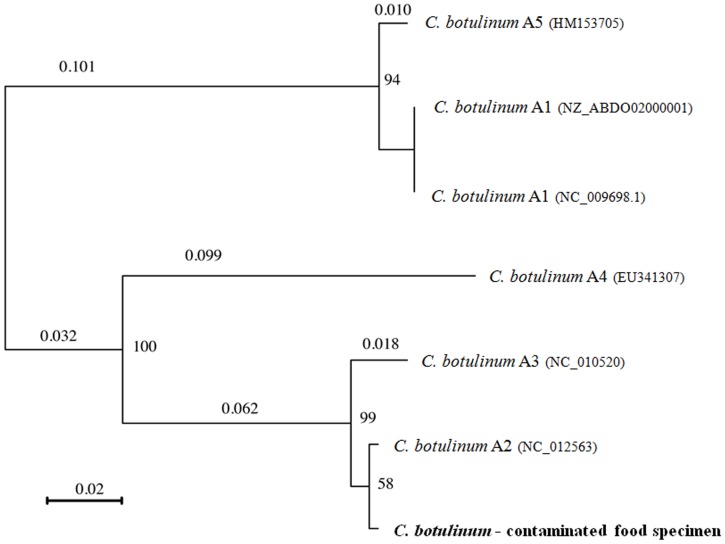
Phylogenetic tree of the *bont/A* sequence retrieved in contaminated food and several toxinotype A strains. A neighbor-joining tree was constructed based on the *bont/A* sequence retrieved by the RMA for the enrichment culture of the *C. botulinum* strain in a naturally contaminated salad specimen, and the corresponding sequences of 6 strains representative of 5 known *C. botulinum* toxinotype A subtypes: strain Hall (A1), NCTC 2916 (A[B]), Kyoto (A2), Loch Maree (A3), 657 (Ba4) and A661222 (A5). Bootstrap values and genetic distance (bar) are shown. The Genbank accession number of each strain is indicated in parentheses. The *bont* sequence obtained from the contaminated salad specimen is marked in bold.

To demonstrate the capacity of the RMA approach to detect *C. botulinum* target sequences in the context of a foodborne outbreak, DNA extracts from naturally contaminated food samples were analysed. Food specimens were collected during the investigation of a *C. botulinum* family outbreak in Corsica [34]. RMA was performed on the DNA extract from a contaminated salad sample, as well as the enrichment culture of the salad sample. For DNA obtained directly from the salad specimen, a total of 286 bacterial prototype sequences were retained, including 3 sequences targeting *C. botulinum* genes (*rrs*, *rpoB* and *ha33*). However, none led to correct taxonomic identification of the *C. botulinum* infecting strain. For the DNA extract obtained from the enrichment culture of the contaminated salad, a total of 15 *C. botulinum* prototype sequences were retained after filtering, with call-rates ranging between 46.7 to 97.7%. In addition, sequences targeting partial *C. perfringens*-specific housekeeping genes (*rpoB*, *gyrA*, and *parC*) and toxin genes (*cpa*, *cpb2* and *pfoR*) were retrieved (call-rates ranging between 70.1 and 98.3%). All filtered sequences led to the retrieval of at least one BLAST hit and corresponding taxonomic identity. The highest scoring BLAST results obtained for *C.perfringens* prototype sequences retrieved by RMA all designated *C. perfringens* strains as taxonomic hit (data not shown). The results of the highest scoring BLAST alignment for each retained *C. botulinum* sequence (total score, coverage and taxonomic identity) are presented in figure 3. Strains belonging to the toxin subtype A2 were recognized as best hit for most of the retrieved sequences of the neurotoxin gene cluster components. In addition, a gene cluster type ha-/orfX+ was identified. Phylogenetic analysis of the retrieved neurotoxin sequence with the corresponding sequences of five strains representative of the different toxin A subtypes (A1 to A5), demonstrated an overall identity of 100% with the neurotoxin sequence of the toxin subtype A2 reference strain. The only mismatches in the multiple alignment were due to unidentified bases (Figure 4).

## Discussion

Although botulism is quite rare in developed countries, recent outbreaks have demonstrated the potential severity of this hazard [35]–[40]. In such a context, rapid identification and characterization of BoNT-producing clostridia in home-made or manufactured food is a priority to stop the spread of botulism infections and trace back the source of contamination [41]–[43]. While identification of BoNT-encoding genes, using rapid nucleic-acid detection tools, is usually satisfactory for molecular risk assessment of BoNT-producing clostridia in food and environmental samples, it is not sufficient to discriminate strains within a toxinotype for source-tracing and molecular epidemiology [16]. For this reason, recent recommendations have stated that new assays should be able to detect variants for all toxinotypes, should be type-specific to determine proper treatment, and should be sensitive to perform risk assessment (NIAID Expert Panel on Botulism Diagnostics, Bethesda Maryland, May 2003). The development of such universal genetic characterization tools is hampered by the great variability of the genomic background, the sequence of the *bont* gene and the arrangement of the BoNT gene cluster of *C. botulinum* strains, and therefore only available for a limited number of strains.

In the present study, we designed and evaluated the performances of a resequencing microarray combined with an automated data approach for the simultaneous detection and characterization of BoNT-producing clostridia. The described RMA, designated PathogenID v2.0, and similar arrays have been proven successful for the characterization of numerous bacteria including *Neisseria meningitidis*, *Streptococcus pyogenes*, *Streptococcus pneumoniae, Bacillus anthracis*, and several species of the *Enterobacteriaceae* family [30], [44]–[46]. For the purpose of this study, prototype sequences of housekeeping genes *rrs* and *rpoB* from clostridia pathogenic for humans and most frequently associated with outbreaks were incorporated in the RMA for identification of clostridial species and lineages [47]–[49]. In addition, sequences from the BoNT gene cluster were included for their involvement in toxicity and usefulness for studying genetic variation [18], [50], [51].

The method was validated with DNA extracts from a representative panel of reference *C. botulinum* strains. The obtained results demonstrated that this approach has the potential to detect neurotoxin-producing *C. botulinum* strains, but also phenotypically related strains, by providing high quality sequence data. The RMA approach reliably detected and characterized the neurotoxin complex genetic determinants for all tested toxino-(sub)types, including bivalent strains. Further analysis of resequencing results showed that targeted sequences were successfully retrieved by the RMA when they presented up to 24.7% divergence with the tiled probes. This data supports the broadness of detection of the RMA, as the divergence of the tiled probes compared to the respective *bont* gene regions from all known toxin A, B, C, D, E and G subtypes, and most toxin F subtypes, was calculated to be inferior to 21%. Although the tiled *bont/F* sequence demonstrated respectively 70.65% and 55.72% with the corresponding gene region of the rare toxin subtypes F5 and F7, the high homology of the additional housekeeping gene and *antp* gene sequences targeted by the RMA should allow detection and correct identification of these strains.

In addition to sequence divergence, differences in DNA extract quality, genomic copy number variation, location of targeted genomic regions and possible secondary structures, as well as the G and C content of the tiled probes and the presence of nonspecific nucleotide stretches could have affected genome amplification and hybridization efficacy, possibly causing reduced base-call rates. Nevertheless, this did not hamper the correct identification and characterization of the analyzed strains.

Subsequently, the pathogenID v2.0 was used to identify *C. botulinum* strains in contaminated food specimens. The analysis of the DNA extract obtained directly from these specimens by the RMA did not permit quality resequencing of the infecting *C. botulinum* strain sequences. These results are due to the presence of numerous cultivable and non-cultivable environmental bacteria naturally present in food, competing with the *C. botulinum* strain during the whole genome amplification step, and subsequently increasing the noise level observed at the time of the analysis. Nevertheless, a 48 h enrichment culture of the contaminated food samples was sufficient to successfully detect and characterize the infecting *C. botulinum* strain as well as a co-cultured *C. perfringens* strain. The results obtained by BLASTing and phylogenetic analysis of the retrieved *bont* sequence, as well as the characterization of the BoNT gene cluster components, classified the strain as a *C. botulinum* toxinotype A2. Moreover, the examination of the taxonomic BLAST hits indicated a close relatedness to strain Mascarpone. As the analysis was performed with a partial sequence of the *bont* gene (200 nt), containing 4% unidentified bases, misclassification of the toxin subtype may have occurred. However, this was unlikely as the retained sequence contained specific determinants found only in *bont/A2* genes, and unidentified residues were located in regions that are not discriminant for subtype differentiation. The *C. botulinum* strain detected by RMA could be isolated from the food sample, and confirmed as subtype A2 by sequencing the specifically PCR-amplified *bont* gene (genbank accession number JQ954970).

The many advantages of using DNA microarrays for characterization of bacteria have been discussed in the past and can be applied to this assay. First, the genetic characterization results can be used to support epidemiological associations in outbreaks involving a large number of samples or those from multiple geographical locations[17]. Secondly, they can be used to trace or confirm the source of an outbreak or to perform environmental risk assessments [52], [53]. In the specific context of botulism, the results provided by DNA microarray can further serve to design specific primers for rapid nucleic acid detection methods, and allow a preliminary differentiation of the strains before applying more laborious genotyping methods such as PFGE [17].

In addition to these advantages, the specific design and properties of high-density RMAs allow the detection of a broad range of strains, including potentially emerging variants [44], [54]. Firstly, the use of closely overlapping 25 nt probe sets in the RMA approach instead of unique probes to cover a target region of 100–500 nt increases the chance for hybridization of divergent target DNA to the array. Indeed, studies using focused or comparative genomic hybridization microarrays have reported a maximum value of 15–18% nucleotide sequence divergence of probe regions for positive microarray detection of *C. botulinum* genomic DNA, as compared to 24.7% for the RMA described here [17]–[19], [24]. In addition, the relatedness between strains can be quantified more precisely by using a sequence-based approach such as the RMA, rather than by analysis of differences in hybridization patterns provided by classic microarrays. This is particularly important in the case of botulism, as the frequent occurrence of gene recombination, insertion and transfer observed between *Clostridium* strains indicate that such events are likely to occur again. In such a case, the sequence information provided by the RMA will be most valuable for the characterization of the emerging variants and the development of improved rapid detection tools. This also emphasizes the need for bacterial culture isolates as well as well-characterized microbial strain collections, in order to monitor the molecular evolution of the strains.

The unique design of the PathogenID v2.0 array allows the simultaneous detection and characterization of 48 sequences targeting the BoNT gene cluster components. These sequences could easily be used in the design of specific RMAs with reduced probe densities by decreasing the number of sequences, thereby diminishing production costs and computing time. Using standard methods to obtain the same level of resolution would imply the use of specific PCRs followed by Sanger sequencing. Although these assays are cheaper as standalone tests, running enough of them to cover the same number of genes with a single sample quickly surpasses the RMA in cost, time and logistical complexity of running the analysis. Moreover these methods rely on sequence-specific primer hybridization for target DNA amplification instead of random hexamers for unbiased whole genome amplification, and may therefore fail to amplify and detect emerging variants. Other technologies with universal coverage could be used, such as high-throughput sequencing, however they are still much more laborious and expensive. Finally, the use of an automated approach allows an unbiased, exhaustive and rapid retrieval of taxonomic results.

In summary, the RMA allowed successful detection and fine characterization of neurotoxin-producing clostridia in both pure and polymicrobial cultures. The RMA approach, combined with the automated filtering and retrieval of taxonomic identities, allowed efficient and accurate subtyping of the neurotoxin and detailed characterization of the BoNT gene cluster components. This assay will be used as a higher echelon component in a diagnostics and surveillance pipeline. Importantly, it will be used in further studies to characterise potential outbreak related strains, but also environment isolates, in order to obtain a better picture of the molecular epidemiology of BoNT-producing clostridia.

## Materials and Methods

### Design of the Pathogen ID v2.0 resequencing microarray (RMA)

The second generation of a broad-spectrum resequencing microarray (RMA) was used in this study: the PathogenID v2.0 array, able to detect 124 bacteria, 126 viruses and 673 genes involved in toxin production, pathogenicity or antibiotic resistance [31]. This array included specific housekeeping genes, namely *rrs* and *rpoB*, for detection and characterization of clostridial species. In addition, it included prototype sequences of each component of the neurotoxin gene cluster from all known toxinotypes, as well as several subtypes. Tiled probes targeting these genes were selected using multiple sequence alignments of genome sequences from various toxin subtypes available in GenBank during the time of the RMA design, in order to select conserved regions within toxinotypes for optimal hybridization efficacy. Additional probes were added to improve the detection of specific subtypes, if strains demonstrated sequence variation within the selected probe region. In addition, the *bont/C* and *bont/D* sequences tiled on the array were specifically designed to maximize the probability of detection of mosaic *C. botulinum* toxinotype C/D or D/C strains, by targeting regions highly homologous to either mosaic *bont/C/D* or *bont/D/C* genes. Finally, 19 additional sequences were tiled on the array for detection of a large panel of clostridial toxins other than neurotoxins.

### Bacterial strains and biological samples analysed

Eleven well-characterized *C. botulinum* strains belonging to toxinotypes A through G served as reference: strains Hall (type A1), 1430-11 [type A5(B′)], NCTC 2916 [type A1(B)], Loch Maree (type A3), BL6 (type B2), 161.08 (type Bf2), NCIB 10658 (type F6), K16 (type E1), 468 (type C1), 1873 (type D) and NCIB 10714 (type G). The characteristics and origin of most strains have been described elsewhere. The *C. botulinum* type A5(B) strain 1430-11 was isolated from contaminated commercial food (pasta). The *C. botulinum* strain 161.08 was isolated from a foodborne botulism case. Pure cultures of *Clostridium* spp. strains were performed as described previously [55]. Genome sequences from reference strains were retrieved from the genbank database. The *bont* sequences for strain 1430-11 (genbank accession numbers KC683799 and KC683800) and strain 161.08 (genbank accession numbers KC471328 and KC471329) were obtained by sanger sequencing of specifically PCR-amplified *bont* genes and deposited in genbank.

Biological samples included a naturally contaminated food specimen (salad) and a 48 h enrichment culture of this food sample in Fortified Cooked Meat Medium (FCMM) at 37°C in anaerobic conditions [56]. Presence of *C.botulinum* toxinotype A in the food samples was confirmed by SYBR green real-time PCR with primers P1646 (5′-TCTTACGCGAAATGGTTATGG-3′) and P1647 (5′-TGCCTGCACCTAAAAGAGGA-3′) for *bont/A* gene, P1648 (5′-CCTGGGCCAGTTTTAAATGA-3′) and P1649 (5′-GCGCCTTTGTTTTCTTGAAC-3′) for *bont/B* gene, P1650 (5′-GTGCCCGAAGGTGAAAATAA-3′) and P1651 (5′-TAATGCTGCTTGCACAGGTT-3′) for *bont/E* gene, P2107 (5′-TGCACAATGAATTTTCAAAACA-3′) and P2108 (5′-TCCAAAAGCATCCATTACTGC-3′) for *bont/F* gene. Real time PCR was performed in a total volume of 25 µl containing 12.5 µl of 2× concentration of iQ SYBR green Supermix (Biorad), 5 pmole of each primer, 5 µl of template DNA and 7.3 µl of DNase-RNase free ditilled water (Gibco). Amplifications were carried out on a CFX96 Real Time System (Biorad) using 96-well microwell plates and according to the following temperature profile: one cycle of 95°C for 10 minutes, 40 cycles of 95°C for 15 sec and 60°C for 30 sec with fluorescence signal capture at the end of each 60°C step, an extension phase of 1 cycle at 95°C for 60 sec, 60°C for 60 sec and 95°C for 60 sec, followed by a default melt (disassociation) stage.

### Extraction and amplification of bacterial DNA

Total genomic DNA was isolated from *C. botulinum* cultures by lysozyme and proteinase K treatment as described previously [57]. DNA extraction from food samples was performed with Powerfood Microbial DNA isolation kit (MoBio Laboratories Inc) according to the manufacturer's recommendations. After extraction, DNA was amplified using the whole genome amplification (WGA) protocol (RepliG Midi kit, Qiagen) as described previously [54].

### Resequencing microarray assay

The amplification products obtained from genomic DNA by WGA were quantified by Quantit BR (Invitrogen) according to the manufacturer's instructions. A recommended amount of target DNA was fragmented and labeled according to the GeneChip Resequencing Assay manual (Affymetrix). The obtained products were coded with unrelated numbers by a non-observer to ensure that the study was performed blindly. The microarray hybridization process was carried out according to the protocol recommended by the manufacturer (Affymetrix). The details and parameter settings for the data analysis (essentially conversion of raw image files obtained from scanning of the microarrays into FASTA files containing the sequences of called bases for each tiled region of the microarray) have been described previously [54]. The base call rate refers to the percentage of base calls generated from the full-length tiled sequence.

### Automated data analysis

A Perl-based program, designated “TaxFinder”, was used for the automated analysis of re-sequencing data provided by PathogenID v2.0. The program read the FASTA file generated for each sample, which contains all the sequences read by the GSEQ software (Affymetrix) from the hybridization results. A filtering process, based on the one described by Malanoski *et* al. [58] was applied to these sequences. For each sequence, TaxFinder considers the Nb first bases of the sequence, Nb defined by the user in the beginning of the experiment and corresponding to the minimal length accepted for the sequence to keep. If the percentage of No-call in this fragment is inferior to an elongation threshold defined by the user, a base is added to the fragment and the percentage of No-call is recalculated. As soon as it is higher that the elongation threshold, the No-call at the ends of the fragment are removed. If the length of the nucleotide fragment is still higher than the minimal threshold established, then it is conserved. If the percentage is higher than the threshold, the fragment is skipped, and the program considers the fragment of size Nb and starting position one base upstream than the precedent fragment. This process is reiterated until the end of the sequence is reached. In this study, sequences that did not contain subsequences with a minimum nucleotide length of 20 nt and a maximum undetermined nucleotide content of 10% were discarded. Filtered sequences individually underwent a blastn analysis to search for sequence homologues in the NCBI nucleotide collection (nr/nt database) with adjusted settings to restrict the search to bacteria entries. Blast + application distributed by NCBI is used for the automated blast research (http://www.ncbi.nlm.nih.gov/books/NBK1763/). The following algorithm parameters were modified: the expected value cut-off was fixed to 100, the minimum word size was set to 7 and the upper limit of displayed descriptions of database sequences per query was decreased to 50. The best BLAST hits were classified according to their total score (the sum of the high-scoring segment pairs) and their corresponding taxonomies were retrieved from the NCBI Taxonomy database. When several hits obtained the highest total score, the script automatically retrieved the taxonomies of the 10 first BLAST hits.

### Phylogenetic analysis

The resulting *bont* sequence obtained from the enrichment culture of the *C. botulinum* strain in a contaminated food specimen was compared with the corresponding *bont* sequences of reference strains from the 5 known *C. botulinum* toxinotype A subtypes (GenBank accessed 21/02/2013). Multiple sequence alignment was performed using the CLC Bio software and checked for accuracy by eye. A neighbor-joining tree of these sequences was constructed using the Jukes-Cantor method with the SeaView v4.2.1 software. The level of support for each node is provided by 100 bootstrap replications.

## References

[1] Sobel J (2005) Botulism. Clin Infect Dis 41: 1167–1173 doi:10.1086/444507 16163636 10.1086/444507

[2] Arnon SS, Schechter R, Inglesby TV, Henderson DA, Bartlett JG, et al (2001) Botulinum Toxin as a Biological Weapon. JAMA 285: 1059–1070 doi:10.1001/jama.285.8.1059 11209178 10.1001/jama.285.8.1059

[3] Villar RG, Elliott SP, Davenport KM (2006) Botulism: The Many Faces of Botulinum Toxin and its Potential for Bioterrorism. Infect Dis Clin North Am 20: 313–327 doi:16/j.idc.2006.02.003 16762741 10.1016/j.idc.2006.02.003

[4] Peck MW (2009) Biology and Genomic Analysis of Clostridium botulinum. Academic Press, Vol. 55: . 183–265, 320. Available: http://www.sciencedirect.com/science/article/pii/S0065291109055039. Accessed 2011 Aug 3. 10.1016/S0065-2911(09)05503-9 19573697

[5] Brüggemann H, Woller A, Mazuet C, Popoff MR (2011) Clostridium botulinum. Genomes of Foodborne and Waterborne Pathogens. Washington DC: Fratamico P., Liu Y., Kathariou S. 185–212.

[6] Smith TJ, Hill KK, Foley BT, Detter JC, Munk AC, et al. (2007) Analysis of the Neurotoxin Complex Genes in Clostridium botulinum A1–A4 and B1 Strains: BoNT/A3,/Ba4 and/B1 Clusters Are Located within Plasmids. PLoS One. 2 .doi:10.1371/journal.pone.0001271. 10.1371/journal.pone.0001271 PMC2092393 18060065

[7] Hill KK, Smith TJ, Helma CH, Ticknor LO, Foley BT, et al (2007) Genetic Diversity among Botulinum Neurotoxin-Producing Clostridial Strains. J Bacteriol 189: 818–832 doi:10.1128/JB.01180-06 17114256 10.1128/JB.01180-06 PMC1797315

[8] Hutson RA, Zhou Y, Collins MD, Johnson EA, Hatheway CL, et al (1996) Genetic Characterization of Clostridium botulinum Type A Containing Silent Type B Neurotoxin Gene Sequences. J Biol Chem 271: 10786–10792 doi:10.1074/jbc.271.18.10786 8631890 10.1074/jbc.271.18.10786

[9] Santos-Buelga JA, Collins MD, East AK (1998) Characterization of the Genes Encoding the Botulinum Neurotoxin Complex in a Strain of Clostridium botulinum Producing Type B and F Neurotoxins. Curr Microbiol 37: 312–318 doi:10.1007/s002849900384 9767710 10.1007/s002849900384

[10] Kirma N, Ferreira JL, Baumstark BR (2004) Characterization of six type A strains of Clostridium botulinum that contain type B toxin gene sequences. FEMS Microbiol Lett 231: 159–164 doi:10.1016/S0378-1097(03)00911-X 14987759 10.1016/S0378-1097(03)00911-X

[11] Lúquez C, Raphael BH, Maslanka SE (2009) Neurotoxin Gene Clusters in Clostridium botulinum Type Ab Strains. Appl Environ Microbiol 75: 6094–6101 doi:10.1128/AEM.01009-09 19684172 10.1128/AEM.01009-09 PMC2753052

[12] Moriishi K, Koura M, Abe N, Fujii N, Fujinaga Y, et al (1996) Mosaic structures of neurotoxins produced from Clostridium botulinum types C and D organisms. Biochim Biophys Acta 1307: 123–126 doi:10.1016/0167-4781(96)00006-1 8679691 10.1016/0167-4781(96)00006-1

[13] Brüggemann H (2005) Genomics of clostridial pathogens: implication of extrachromosomal elements in pathogenicity. Curr Opin Microbiol 8: 601–605 doi:16/j.mib.2005.08.006 16125440 10.1016/j.mib.2005.08.006

[14] Smith TJ, Lou J, Geren IN, Forsyth CM, Tsai R, et al (2005) Sequence Variation within Botulinum Neurotoxin Serotypes Impacts Antibody Binding and Neutralization. Infect Immun 73: 5450–5457 doi:–10.1128/IAI.73.9.5450–5457.2005 16113261 10.1128/IAI.73.9.5450-5457.2005 PMC1231122

[15] Hill KK, Xie G, Foley BT, Smith TJ, Munk AC, et al (2009) Recombination and insertion events involving the botulinum neurotoxin complex genes in Clostridium botulinum types A, B, E and F and Clostridium butyricum type E strains. BMC Biol 7: 66–66 doi:10.1186/1741-7007-7-66 19804621 10.1186/1741-7007-7-66 PMC2764570

[16] Fach P, Fenicia L, Knutsson R, Wielinga PR, Anniballi F, et al (2011) An innovative molecular detection tool for tracking and tracing Clostridium botulinum types A, B, E, F and other botulinum neurotoxin producing Clostridia based on the GeneDisc cycler. Int J Food Microbiol 145: S145–S151 doi:16/j.ijfoodmicro.2010.04.006 20471128 10.1016/j.ijfoodmicro.2010.04.006

[17] Raphael BH, Joseph LA, McCroskey LM, Lúquez C, Maslanka SE (2010) Detection and differentiation of Clostridium botulinum type A strains using a focused DNA microarray. Mol Cell Probes 24: 146–153 doi:16/j.mcp.2009.12.003 20056143 10.1016/j.mcp.2009.12.003

[18] Carter AT, Paul CJ, Mason DR, Twine SM, Alston MJ, et al (2009) Independent evolution of neurotoxin and flagellar genetic loci in proteolytic Clostridium botulinum. BMC Genomics 10: 115–115 doi:10.1186/1471-2164-10-115 19298644 10.1186/1471-2164-10-115 PMC2674064

[19] Sebaihia M, Peck MW, Minton NP, Thomson NR, Holden MTG, et al (2007) Genome sequence of a proteolytic (Group I) Clostridium botulinum strain Hall A and comparative analysis of the clostridial genomes. Genome Res 17: 1082–1092 doi:10.1101/gr.6282807 17519437 10.1101/gr.6282807 PMC1899119

[20] Umeda K, Seto Y, Kohda T, Mukamoto M, Kozaki S (2010) A novel multiplex PCR method for Clostridium botulinum neurotoxin type A gene cluster typing. Microbiol Immunol 54: 308–312 doi:10.1111/j.1348-0421.2010.00213.x 20536728 10.1111/j.1348-0421.2010.00213.x

[21] Umeda K, Seto Y, Kohda T, Mukamoto M, Kozaki S (2009) Genetic Characterization of Clostridium botulinum Associated with Type B Infant Botulism in Japan. J Clin Microbiol 47: 2720–2728 doi:10.1128/JCM.00077-09 19571018 10.1128/JCM.00077-09 PMC2738102

[22] Leclair D, Pagotto F, Farber JM, Cadieux B, Austin JW (2006) Comparison of DNA Fingerprinting Methods for Use in Investigation of Type E Botulism Outbreaks in the Canadian Arctic. J Clin Microbiol 44: 1635–1644 doi:–10.1128/JCM.44.5.1635–1644.2006 16672387 10.1128/JCM.44.5.1635-1644.2006 PMC1479196

[23] Fillo S, Giordani F, Anniballi F, Gorgé O, Ramisse V, et al (2011) Clostridium botulinum Group I Strain Genotyping by 15-Locus Multilocus Variable-Number Tandem-Repeat Analysis. J ClinMicrobiol 49: 4252–4263 doi:10.1128/JCM.05396-11 10.1128/JCM.05396-11 PMC3232984 22012011

[24] Lindström M, Hinderink K, Somervuo P, Kiviniemi K, Nevas M, et al (2009) Comparative Genomic Hybridization Analysis of Two Predominant Nordic Group I (Proteolytic) Clostridium botulinum Type B Clusters. Appl Environ Microbiol 75: 2643–2651 doi:10.1128/AEM.02557-08 19270141 10.1128/AEM.02557-08 PMC2681723

[25] Han T, Chang CW, Kwekel JC, Chen Y, Ge Y, et al (2012) Characterization of whole genome amplified (WGA) DNA for use in genotyping assay development. BMC Genomics 13: 217 doi:10.1186/1471-2164-13-217 22655855 10.1186/1471-2164-13-217 PMC3403925

[26] Erlandsson L, Rosenstierne MW, McLoughlin K, Jaing C, Fomsgaard A (2011) The Microbial Detection Array Combined with Random Phi29-Amplification Used as a Diagnostic Tool for Virus Detection in Clinical Samples. PLoS One 6: e22631 doi:10.1371/journal.pone.0022631 21853040 10.1371/journal.pone.0022631 PMC3154197

[27] Bouzid M, Heavens D, Elwin K, Chalmers RM, Hadfield SJ, et al (2010) Whole genome amplification (WGA) for archiving and genotyping of clinical isolates of Cryptosporidium species. Parasitology 137: 27–36 doi:10.1017/S0031182009991132 19765343 10.1017/S0031182009991132

[28] Gadkar V, Rillig MC (2005) Suitability of genomic DNA synthesized by strand displacement amplification (SDA) for AFLP analysis: genotyping single spores of arbuscular mycorrhizal (AM) fungi. J Microbiol Methods 63: 157–164 doi:10.1016/j.mimet.2005.03.009 15936100 10.1016/j.mimet.2005.03.009

[29] Berthet N, Reinhardt AK, Leclercq I, van Ooyen S, Batéjat C, et al (2008) Phi29 polymerase based random amplification of viral RNA as an alternative to random RT-PCR. BMC Mol Biol 9: 77 doi:10.1186/1471-2199-9-77 18771595 10.1186/1471-2199-9-77 PMC2535778

[30] Zwick ME, Mcafee F, Cutler DJ, Read TD, Ravel J, et al (2005) Microarray-based resequencing of multiple Bacillus anthracis isolates. Genome Biol 6: R10–R10 doi:10.1186/gb-2004-6-1-r10 15642093 10.1186/gb-2004-6-1-r10 PMC549062

[31] Dacheux L, Berthet N, Dissard G, Holmes EC, Delmas O, et al (2010) Application of Broad-Spectrum Resequencing Microarray for Genotyping Rhabdoviruses. J Virol 84: 9557–9574 doi:10.1128/JVI.00771-10 20610710 10.1128/JVI.00771-10 PMC2937603

[32] Wang Z, Malanoski AP, Lin B, Kidd C, Long NC, et al (2008) Resequencing microarray probe design for typing genetically diverse viruses: human rhinoviruses and enteroviruses. BMC Genomics 9: 577–577 doi:10.1186/1471-2164-9-577 19046445 10.1186/1471-2164-9-577 PMC2607299

[33] Lin B, Malanoski AP (2009) Resequencing Arrays for Diagnostics of Respiratory Pathogens. In: Dufva M, DNA Microarrays for Biomedical Research. Totowa, NJ: Humana Press ,Vol. 529 . 231–257. Available: http://www.springerlink.com/content/q1102392j0782ql1/#section=55300&page=1. Accessed 2011 Aug 9. 10.1007/978-1-59745-538-1_15 PMC7176175 19381976

[34] Oriot C, D'Aranda E, Castanier M, Glaizal M, Galy C, et al (2011) One collective case of type A foodborne botulism in Corsica. Clin Toxicol 49: 752–754 doi:10.3109/15563650.2011.606222 10.3109/15563650.2011.606222 21970773

[35] Lai LS, Wang YM, Lin CH (2011) Foodborne botulinum type E intoxication associated with dried bean curd: first case report in Taiwan. Acta Neurol Taiwan 20: 138–141. 21739393

[36] Jalava K, Selby K, Pihlajasaari A, Kolho E, Dahlsten E, et al. (2011) Two cases of food-borne botulism in Finland caused by conserved olives, October 2011. Euro Surveill. 16: : 20034. Available: http://www.eurosurveillance.org/ViewArticle.aspx?ArticleId=20034. Accessed 2011 Dec. 27. 10.2807/ese.16.49.20034-en 22172330

[37] Browning LM, Prempeh H, Little C, Houston C, Grant K, et al. (2011) An outbreak of food-borne botulism in Scotland, United Kingdom, November 2011. Euro Surveill. 16: : 20036. Available: http://www.eurosurveillance.org/ViewArticle.aspx?ArticleId=20036. Accessed 2011 Dec 27. 10.2807/ese.16.49.20036-en 22172331

[38] Pingeon JM, Vanbockstael C, Popoff MR, King LA, Deschamps B, et al. (2011) Two outbreaks of botulism associated with consumption of green olive paste, France, September 2011. Euro Surveill. 16: : 20035. Available: http://www.eurosurveillance.org/ViewArticle.aspx?ArticleId=20035. Accessed 2011 Dec 27. 10.2807/ese.16.49.20035-en 22172329

[39] Daminelli P, De Nadai V, Bozzo G, Finazzi G, Oliverio E, et al (2011) Two unlinked cases of foodborne botulism in Italy at the beginning of 2010. New Microbiol 34: 287–290. 21811749

[40] Date K, Fagan R, Crossland S, MacEachern D, Pyper B, et al (2011) Three Outbreaks of Foodborne Botulism Caused by Unsafe Home Canning of Vegetables Ohio and Washington, 2008 and 2009. J Food Prot 74: 2090–2096 doi:10.4315/0362-028X.JFP-11-128 22186049 10.4315/0362-028X.JFP-11-128

[41] Case definitions for infectious conditions under public health surveillance. Centers for Disease Control and Prevention (1997) MMWR Recomm Rep 46: 1–55. 9148133

[42] Therre H. (1999) Botulism in the European Union. Euro Surveill.. 4: : 2–7. Available:http://www.eurosurveillance.org/ViewArticle.aspx?ArticleId=48. Accessed 28 December 2011. 10.2807/esm.04.01.00048-en 12631917

[43] Lindström M, Korkeala H (2006) Laboratory Diagnostics of Botulism. Clin Microbiol Rev 19: 298–314 doi:–10.1128/CMR.19.2.298–314.2006 16614251 10.1128/CMR.19.2.298-314.2006 PMC1471988

[44] Pandya GA, McEllistrem MC, Venepally P, Holmes MH, Jarrahi B, et al. (2011) Monitoring the Long-Term Molecular Epidemiology of the Pneumococcus and Detection of Potential “Vaccine Escape” Strains. PLoS One 6 . doi:10.1371/journal.pone.0015950. 10.1371/journal.pone.0015950 PMC3018475 21264340

[45] Corless CE, Kaczmarski E, Borrow R, Guiver M (2008) Molecular Characterization of Neisseria meningitidis Isolates Using a Resequencing DNA Microarray. J Mol Diagn 10: 265–271 doi:10.2353/jmoldx.2008.070152 18372424 10.2353/jmoldx.2008.070152 PMC2329792

[46] Davignon L, Walter EA, Mueller KM, Barrozo CP, Stenger DA, et al (2005) Use of Resequencing Oligonucleotide Microarrays for Identification of Streptococcus pyogenes and Associated Antibiotic Resistance Determinants. J Clin Microbiol 43: 5690–5695 doi:–10.1128/JCM.43.11.5690–5695.2005 16272506 10.1128/JCM.43.11.5690-5695.2005 PMC1287778

[47] Hutson RA, Thompson DE, Lawson PA, Schocken-Itturino RP, Böttger EC, et al (1993) Genetic interrelationships of proteolytic Clostridium botulinum types A, B, and F and other members of the Clostridium botulinum complex as revealed by small-subunit rRNA gene sequences. Antonie Van Leeuwenhoek 64: 273–283. 8085790 10.1007/BF00873087

[48] Pourshaban M, Franciosa G, Fenicia L, Aureli P (2002) Taxonomic identity of type E botulinum toxin-producing Clostridium butyricum strains by sequencing of a short 16S rDNA region. FEMS Microbiol Lett 214: 119–125. 12204382 10.1111/j.1574-6968.2002.tb11334.x

[49] Sasaki Y, Takikawa N, Kojima A, Norimatsu M, Suzuki S, et al (2001) Phylogenetic positions of Clostridium novyi and Clostridium haemolyticum based on 16S rDNA sequences. Int J Syst Evol Microbiol 51: 901–904. 11411712 10.1099/00207713-51-3-901

[50] Franciosa G, Floridi F, Maugliani A, Aureli P (2004) Differentiation of the gene clusters encoding botulinum neurotoxin type A complexes in Clostridium botulinum type A, Ab, and A(B) strains. Appl Environ Microbiol 70: 7192–7199 doi:–10.1128/AEM.70.12.7192–7199.2004 15574917 10.1128/AEM.70.12.7192-7199.2004 PMC535171

[51] Raphael BH, Luquez C, McCroskey LM, Joseph LA, Jacobson MJ, et al (2008) Genetic Homogeneity of Clostridium botulinum Type A1 Strains with Unique Toxin Gene Clusters. Appl Environ Microbiol 74: 4390–4397 doi:10.1128/AEM.00260-08 18502928 10.1128/AEM.00260-08 PMC2493146

[52] Leski TA, Malanoski AP, Gregory MJ, Lin B, Stenger DA (2011) Application of a Broad-Range Resequencing Array for Detection of Pathogens in Desert Dust Samples from Kuwait and Iraq. Appl Environ Microbiol 77: 4285–4292 doi:10.1128/AEM.00021-11 21571877 10.1128/AEM.00021-11 PMC3127696

[53] Leski TA, Lin B, Malanoski AP, Wang Z, Long NC, et al. (2009) Testing and Validation of High Density Resequencing Microarray for Broad Range Biothreat Agents Detection. PLoS One 4 . doi:10.1371/journal.pone.0006569. 10.1371/journal.pone.0006569 PMC2719057 19668365

[54] Berthet N, Dickinson P, Filliol I, Reinhardt AK, Batejat C, et al (2008) Massively parallel pathogen identification using high-density microarrays. Microb Biotechnol 1: 79–86. 21261824 10.1111/j.1751-7915.2007.00012.x PMC3864434

[55] Fach P, Micheau P, Mazuet C, Perelle S, Popoff M (2009) Development of real-time PCR tests for detecting botulinum neurotoxins A, B, E, F producing Clostridium botulinum, Clostridium baratii and Clostridium butyricum. J Appl Microbiol 107: 465–473 doi:–10.1111/j.1365–2672.2009.04215.x 19291235 10.1111/j.1365-2672.2009.04215.x

[56] Takeda M, Tsukamoto K, Kohda T, Matsui M, Mukamoto M, et al (2005) Characterization of the neurotoxin produced by isolates associated with avian botulism. Avian Dis 49: 376–381. 16252491 10.1637/7347-022305R1.1

[57] Dineen SS, Bradshaw M, Johnson EA (2003) Neurotoxin Gene Clusters in Clostridium botulinum Type A Strains: Sequence Comparison and Evolutionary Implications. Curr Microbiol 46: 345–352 doi:10.1007/s00284-002-3851-1 12732962 10.1007/s00284-002-3851-1

[58] Malanoski AP, Lin B, Wang Z, Schnur JM, Stenger DA (2006) Automated identification of multiple micro-organisms from resequencing DNA microarrays. Nucleic Acids Res 34: 5300–5311 doi:10.1093/nar/gkl565 17012284 10.1093/nar/gkl565 PMC1636417

[59] Franciosa G, Maugliani A, Floridi F, Aureli P (2006) A novel type A2 neurotoxin gene cluster in Clostridium botulinum strain Mascarpone. FEMS Microbiol Lett 261: 88–94 doi:10.1111/j.1574-6968.2006.00331.x 16842364 10.1111/j.1574-6968.2006.00331.x

